# High ATF4 Expression Is Associated With Poor Prognosis, Amino Acid Metabolism, and Autophagy in Gastric Cancer

**DOI:** 10.3389/fonc.2021.740120

**Published:** 2021-12-17

**Authors:** Mingliang Wang, Yida Lu, Huizhen Wang, Youliang Wu, Xin Xu, Yongxiang Li

**Affiliations:** General Surgery Department, The First Affiliated Hospital of Anhui Medical University, Hefei, China

**Keywords:** gastric cancer, ATF4, activating transcription factor 4, nomogram, metabolism, autophagy

## Abstract

**Background:**

The role of activating transcription factor 4 (ATF4) underlying gastric cancer (GC) remains unclear. The purpose of this study was to investigate the expression levels and biological functions of ATF4 in GC.

**Methods:**

Expression of ATF4 was detected by quantitative PCR (qPCR), Western blotting, and immunohistochemistry. Cox regression was used for survival analysis and the construction of the nomogram. Immunofluorescence was used to identify the intracellular localization of ATF4. Knockdown and overexpression of ATF4 in GC cells followed by wound healing and Transwell assays, EdU and Calcein-AM/propidium iodide (PI) staining, and cell cycle detection were performed to examine its function *in vitro*. Transmission electron microscopy was performed to assess the autophagy levels upon ATF4 silencing. Kyoto Encyclopedia of Genes and Genomes (KEGG) analysis and gene set enrichment analysis (GSEA) were used to determine gene enrichment. SPSS 22.0 software, GraphPad Prism 7.0, and R version 3.6.1 were used for statistical analysis.

**Results:**

ATF4 expression was upregulated in GC cells and tissues compared with corresponding normal tissues. Survival analysis suggested that a high ATF4 expression was strongly associated with worse overall survival (OS) of GC patients (*p* < 0.001). The nomogram and the receiver operating characteristic (ROC) curves demonstrated that ATF4 was a highly sensitive and specific prognostic marker of GC [C-index = 0.797, area under the ROC curve (AUC) of 3-year OS = 0.855, and AUC of 5-year OS = 0.863]. In addition, ATF4 knockdown inhibited the cell proliferation, migration, invasion, and cell cycle progression of GC cells *in vitro*, while overexpression of ATF4 exerted the opposite effects. Bioinformatics analysis showed that ATF4 could promote GC progression possibly by regulating asparagine (Asn) metabolism and autophagy pathways. Further experiments indicated that ATF4 expression was significantly positively correlated with ASNS expression. The inhibition of cell clone formation in Asn-deprived conditions was more significant in the shATF4 group. Finally, we found that ATF4 promoted autophagy through regulating the mTORC1 pathway in GC cells.

**Conclusion:**

These findings suggested that ATF4 can significantly promote GC development and serve as an independent prognostic factor for GC.

## Introduction

Gastric cancer (GC) is the fifth most commonly diagnosed cancer and the second leading cause of cancer-related deaths worldwide according to the Global Cancer Statistics 2018 (1). Recently, although biological agents, anti-angiogenic therapeutic and immunotherapeutic agents, have been used in advanced GC (2–4), the median survival time of these patients is less than 12 months (5). Therefore, elucidating the molecular mechanisms underlying GC progression is valuable for the identification of potential targets and the development of therapeutic methods for GC.

The activating transcription factor (activating transcription factor/cAMP response element binding protein, ATF/CREB) family shares a conserved basic-region leucine zipper domain and was first named in 1987 (6). To date, over 20 ATF/CREB members have been described in mammals, and several of these members serve important roles in cancer progression (7). For example, ATF2 knockdown inhibits cell growth and increases sensitivity toward chemotherapy in pancreatic cancer (8). ATF3 acts as a key regulator of immune responses and metabolic homeostasis in various cancer types (9). ATF6 plays a crucial role in cell growth, migration, and apoptosis in cervical cancer (10).

As an important member of the ATF/CREB family, activating transcription factor 4 (ATF4) regulates tumor progression mainly through the following aspects. Firstly, ATF4 may promote cancer progression by regulating the homeostasis of several metabolites, including amino acid and glucose metabolism (11–13). For example, the absence of P62 in stromal cells leads to the upregulation of ATF4 to sustain asparagine (Asn)-mediated tumor growth (11). ATF4 also allows CD4^+^ T cells to adapt to oxidizing environments and amino acid starvation by enhancing the *de novo* synthesis of amino acids (12). As an important regulatory factor of amino acid metabolism, mTORC1 is closely related to ATF4 (14). ATF4 could promote protein and glutathione synthesis through mTORC1-dependent activation (15). In addition, ATF4 modulates glucose metabolism by favoring embryonic stem cell phosphatase expression in osteoblasts (13). Secondly, silencing ATF4 significantly inhibits autophagy-dependent cell proliferation in acute myeloid leukemia and other cancer types (16, 17). Moreover, ATF4 has been identified to bind a range of oncoproteins, including eIF2 and c-Myc (17, 18). Thirdly, ATF4 promotes neoplastic transformation and tumor metastasis by suppressing cell senescence and anoikis, respectively (19, 20). As a stress-induced transcription factor, ATF4 could protect transformed cells by inducing the expression of heme oxygenase-1 (20). Taken together, these findings suggested that ATF4 may play an important role in cellular processes in tumors.

Nevertheless, the expression and biological functions of ATF4 in GC remain unknown. In the present study, we tried to address the above issues through immunohistochemistry using self-generated tissue microarray, molecular biology experiments, and cellular functional assays. The purpose of this study was to provide a new potential target for GC therapy.

## Materials and Methods

### Patient and Tissue Microarray

This study was approved by Anhui Medical University Ethics Committee. The ethical code is 20180323. In the present study, 115 GC tissues and 24 randomly selected corresponding adjacent normal tissues were obtained from the General Surgery Department of The First Affiliated Hospital of Anhui Medical University between 2006 and 2008 for the construction of the tissue microarray (TMA). The follow-up period was between 3 and 74 months. All GC tissues were pathologically confirmed and staged by pathologists according to the tumor node metastasis (TNM) staging system and the American Joint Committee on Cancer (7th edition) (21). The following clinical factors of GC patients were collected: age, gender, grade, tumor size, tumor site, tumor depth, tumor differentiation, lymph node status, and TNM stage. The clinicopathological parameters of the enrolled patients are shown in **Table 1**.

**Table 1:** Clinicopathological parameters and Cox regression analysis of the enrolled patients (*n* = 115).

Variables	No. of patients	Univariate analysis		Multivariate analysis	
		HR (95%CI)	*p*-value	HR (95%CI)	*p*-value
Gender					
Male	89	Reference		Reference	
Female	26	1.435 (0.700–2.942)	0.325	–	–
Age (years)					
<61	57	Reference		Reference	
≥61	58	1.017 (0.594–1.743)	0.950	–	–
Tumor grade					
I/II	57	Reference		Reference	
III	58	0.576 (0.332–0.999)	0.05	1.854 (1.056–3.258)	**0.032**
Tumor diameter (cm)					
<6	79	Reference		Reference	
≥6	36	1.572 (0.898–2.752)	0.113	–	–
Differentiation					
High/middle	32	Reference		Reference	
Low	83	0.323 (0.146–0.716)	0.005	2.574 (1.154–5.714)	**0.021**
Location					
Upper	62	Reference		Reference	
Middle	20	1.525 (0.766–3.037)	0.230	–	–
Lower	33	1.755 (0.760–4.050)	0.188	–	–
Depth of invasion					
T1/T2	25	Reference		Reference	
T3/T4		0.101 (0.024–0.414)	0.001	2.900 (0.404–22.792)	0.290
Lymph node metastasis					
No	39	Reference		Reference	
Yes	76	4.823 (2.173–10.703)	0.000	1.704 (0.127–22.792)	0.687
TNM stage					
I/II	50	Reference		Reference	
III/IV	65	0.148 (0.069–0.315)	0.000	2.599 (0.176–38.458)	0.487
ATF4 expression					
Low	58	Reference		Reference	
High	57	0.281 (0.154–0.513)	0.000	2.955 (1.596–5.472)	**0.001**

HR, hazard ratio; CI, confidence interval; ATF4, activating transcription factor 4. Entries in bold: independent risk factors for prognosis.

### Cell Culture

The normal gastric epithelial cell line GES-1 and the GC cell lines AGS, SGC7901, MGC803, and HGC27 were obtained from GeneChem (Shanghai, China). These cells were tested for mycoplasma every month to ensure that they are free of mycoplasma contamination. The method is as follows: the nucleus was stained with DAPI and observed under an oil microscope. Mycoplasma contamination is characterized by radiating or satellite-shaped spots around the nucleus (22). All cell lines were cultured in RPMI-1640 medium (Corning, Corning, NY, USA), supplemented with 10% fetal bovine serum (FBS) (Clark Bioscience, Richmond, VA, USA), penicillin (100 U/ml), and streptomycin (100 μg/ml) (HyClone, Logan, UT, USA) in a humidified atmosphere at 37°C with 5% CO_2_. RPMI-1640 medium lacking Asn was customized by Meilun Biotechnology Co. (Dalian, China). For Asn deprivation assays, the cells were plated overnight in complete RPMI-1640 and subsequently transferred into Asn-free medium after a brief wash with phosphate-buffered saline (PBS).

### RNA Isolation and qPCR Assays

Total RNA was extracted from six paired GC tumors and corresponding adjacent normal tissues and cells using TRIzol Reagent (Invitrogen, Carlsbad, CA, USA). For real-time PCR, we first obtained the cDNA by reverse transcription using ReverTra Ace qPCR RT Master Mix (Toyobo, Osaka, Japan). Then, quantitative PCR (qPCR) was performed using SYBR Green Mix (Toyobo) on the Agilent Mx3000P qPCR Platform (Agilent, Santa Clara, CA, USA). The specific primer sequences were as follows: ATF4-F: 5′-GTCCTCCACTCCAGATCATTCC-3′; ATF4-R: 5′-AGGACTCT GGGCTCATACAGAT-3′. *GAPDH* served as the reference gene, and the primer sequences were as follows: GAPDH-F: 5′-ATCA AGAAGGTGGTGAAGCAGG-3′; GAPDH-R: 5′-CGTCAAAGGTGGAGGAGT GG-3′. The 2^−△△Ct^ equation was used to calculate the relative ATF4 mRNA expression.

### Western Blotting

Total proteins were extracted using the M-per protein lysis buffer containing phosphatase and protease inhibitors (BBI Life Sciences Corporation, Shanghai, China) according to the manufacturer’s instructions. The cell proteins were separated by denaturing SDS-PAGE and the separated proteins transferred onto PVDF membranes (Millipore, Burlington, MA, USA). Subsequently, the membranes were blocked using 5% skimmed milk in PBS for 1 h, after which primary antibodies against ATF4 (1:1,000, cat. #381426; ZenBio, Chengdu, China), ASNS (1:1,000, cat. #sc-365809; Santa Cruz Biotechnology, Santa Cruz, CA, USA), LC3B (1:1,000, cat. #ab192890; Abcam, Cambridge, UK), P62 (1:1,000, cat. # ab207305; Abcam), P70S6K [1:1,000, cat. #2708T; Cell Signaling Technology (CST), Danvers, MA, USA], p-P70S6K (1:1,000, cat. #9862T; CST), 4EBP1 (1:1,000, cat. #9644T; CST), p-4EBP1 (1:1,000, cat. #9862T; CST), JNK1/2/3 (1:1,000, cat. #AF6318; Affinity, Jiangsu, China), p-JNK1/2/3 (Tyr185) (1:1,000, cat. #AF3320; Affinity), and GAPDH (1:2,500, cat. #7074T; CST) were correspondingly added to the blots and incubated overnight at 4°C. The membranes were visualized using enhanced chemiluminescence (ECL) (Bridgen, Beijing, China) and imaged using the Tanon-5200 Multi-Platform (Shanghai, China).

### Immunofluorescence Staining

Immunofluorescence (IF) staining was performed to confirm the subcellular localization of proteins. The detailed experimental steps are as follows: indicated cells were seeded in a 24-well plate with cell slides overnight. The next day, GC cells were fixed with 4% formaldehyde. Subsequently, the cell slides were blocked using 5% bovine serum albumin (BSA) at 26°C for 1 h. Afterwards, they were incubated with ATF4 antibody (1:100, cat. #381426; ZenBio) or LC3B antibody (1:100, cat. #ab192890; Abcam) and then incubated overnight at 4°C. On the second day, the cell slides were incubated with fluorescent secondary antibody (1:250, cat. #A11012; Thermo Fisher Scientific, Waltham, MA, USA) for 1 h at room temperature. Finally, they were stained with DAPI (1:100, cat. #D9542; Sigma, Roedermark, Germany) for 15 min and pictures were recorded using a confocal laser microscope (LSM800; Carl Zeiss, Jena, Germany).

### Immunohistochemistry

Immunohistochemical (IHC) staining was conducted as described previously (23) to analyze the expressions of ATF4 (1:100, ZenBio) and ASNS (1:150, Affinity). The protein level expression in each TMA was evaluated by two pathologists blinded to patients’ clinical information. The IHC scores were calculated based on both the staining intensity and the staining area. The staining intensity was graded on a scale of 0 to 3 points (0 point, negative; 1 point, light yellow; 2 points, claybank; and 3 points, tan). The staining area was scored as 0 (0%), 1 (1%–25%), 2 (26%–50%), 3 (51%–75%), or 4 (76%–100%) based on the percentage of positively stained mucosal cells. The final score was determined as the product of the staining intensity and staining area. A final score of ≤4 points (0, 1, 2, 3, and 4) for a tissue array was considered negative or low expression, and a final score of ≥5 points (6, 8, 9, and 12) was considered high expression.

### Cell Lentivirus Infection

Three human ATF4 small hairpin RNAs (shRNAs) and an overexpression lentiviral ATF4 construct (NM_001675) were purchased from GeneChem (Shanghai, China). AGS, MGC803, and HGC27 cells were infected with lentiviral shRNA against ATF4 (GV248-ATF4; element sequence: hU6–MCS–ubiquitin–EGFP–IRES–puromycin) or the control lentivirus construct (GV248). The SGC7901 cell line was infected with ATF4 overexpression lentivirus (GV341-ATF4; element sequence: Ubi–MCS–3FLAG–SV40–puromycin) or control lentivirus (GV341). For cell lentivirus infection, indicated cells were seeded in 12-well plates (1 × 10^5^ cells/well). Then, ATF4 shRNAs and overexpression lentivirus were added into the plates with lentivirus particles at a multiplicity of infection (MOI) about 10. Stable polyclonal GC cell lines were established after about 2 weeks of antibiotic selection (puromycin, 2 mg/ml). Western blotting (WB) was performed to determine the efficiency of knockdown and overexpression. The shRNA sequences were as follows: sh#1: 5′-CAGAUUGGAUG UUGGAGAATT-3′; sh#2: 5′-GCCUAGGUCUCUUAGAUGAUUTT-3′; and sh#3: 5′-CUGCUUACGUUGCCAUG AUTT-3′.

### Wound-Healing and Transwell Assays

As previously reported (24), wound-healing and Transwell assays were performed to assess the cell migration and invasion abilities, respectively. For the wound-healing assays, GC cells were seeded in six-well plates (6 × 10^5^ cells/well). The next day, when the cells had adhered to the bottom and the confluency was 90%, RPMI-1640 was replaced with FBS-free RPMI-1640 after creating linear wounds. Celldiscoverer 7 Live Cell Station was used to record the progress of wound closure at both 0 and 48 h. The images were analyzed quantitatively using ImageJ software. Three images were used in each group for measurements. For each image, the wound widths at both 0 and 48 h were measured at several intervals. The migration rate (in percent) was calculated by dividing the migration distance by the original width.

For the Transwell invasion assays, the Transwell chamber was placed into a 24-well plate. Approx. 100 μl diluted Matrigel matrix (1 mg/ml) (BD Biosciences, Shanghai, China) was added to the upper chamber and left to set for 5 h in an incubator. Thereafter, infected GC cells in 100 μl serum-free medium were added to the upper chamber and 650 μl RPMI-1640 medium supplemented with 20% FBS was added to the lower chamber. Thirty-six hours later, the Transwell chamber was removed and the cells were fixed with 4% paraformaldehyde for 20 min and stained with 1% crystal violet for 15 min. Then, the cells on the upper surface of the chamber were flushed and recorded using a Leica microscope (DMI1; Wetzlar, Germany). Three images in each group were analyzed quantitatively by ImageJ software. Finally, the rate of invasion of migrating cells was compared between the experimental group and the control group.

### EdU Assay

BeyoClick™ EdU Cell Proliferation Kit (C0078S; Beyotime, Shanghai, China) was used to conduct the 5-ethynyl-2′-deoxyuridine (EdU) assay. Indicated cells were seeded in 24-well plates (1 × 10^5^ cells/well) overnight. Then, 10 µM EdU was added into the medium and the cells were incubated for 2 h at 37°C/5% CO_2._ EdU-labeled cells were incubated with Click Addictive Solution for 30 min at room temperature. The cells were then observed and imaged using the Leica microscope after staining with Hoechst33342 (1:1,000) for 10 min.

### Calcein-AM/Propidium Iodide Staining and Cell Cycle Assays

Cell death was assessed by Calcein-AM/PI staining. Briefly, infected cells were seeded on a cell slider until the cells adhered to the bottom. On the next day, the cells were stained using a solution of 2 μM Calcein-AM and 4.5 μM PI per well at 37°C for 30 min. Finally, the images of stained cells were immediately acquired using the Leica fluorescence microscope (LSM800; Carl Zeiss). Three low-power fields were randomly selected for each group, and the apoptotic cells were quantified by ImageJ software.

For cell cycle assays, after cell harvesting and centrifugation, 75% alcohol was used to fix the treated cells overnight. On the next day, the supernatant was removed after centrifugation at room temperature. Approx. 500 μl PI (50 μg/ml) was added to the Eppendorf tube and dyed for 30 min. Finally, cell cycle changes were detected using the CytoFLEX Flow Cytometry Platform (Beckman Coulter, Brea, CA, USA).

### Colony Formation Assay

Briefly, about 500 infected GC cells were seeded in six-well plates. After culturing at 37°C for about 2 weeks, 4% paraformaldehyde was added to fix the colonies for 20 min. Then, 1% crystal violet was added for staining after discarding paraformaldehyde. The colonies were counted using a digital camera. All experiments were repeated at least three times. Statistical significance was determined by the Mann–Whitney *U*-test.

### Bioinformatics Analysis

RNA-sequencing (RNA-seq) datasets were downloaded from The Cancer Genome Atlas (TCGA) website (https://portal.gdc.cancer.gov/). R version 3.6.1 was used to screen differentially expressed genes between the high-ATF4-expression group and the low-ATF4-expression group. Adjusted *p*-value <0.05 and |logFC| > 0.5 were considered to have significant difference. A heatmap was used to visualize the differentially expressed genes that may be regulated by ATF4. All the differentially expressed genes were queried in Kyoto Encyclopedia of Genes and Genomes (KEGG) (http://www.kegg.jp/) analysis to identify the corresponding enriched pathways. KEGG is a knowledge base for systematic analysis of gene functions. This database integrates genome sequences and other high-throughput data to facilitate the analysis of the molecular function of genes and proteins (25). Therefore, more comprehensive information on the ATF4-related pathways can be obtained with KEGG analysis. Gene set enrichment analysis (GSEA) was conducted to identify the specific pathways regulated by ATF4. GSEA was routinely used to analyze and interpret coordinate pathway-level changes in bioinformatics analysis (26). When the ATF4-related pathways have been identified using KEGG, GSEA was subsequently conducted to reanalyze the specific pathways so as to elucidate the relationship between ATF4 and these pathways.

### Transmission Electron Microscopy

Briefly, 10^7^ indicated cells were seeded in 100-mm Petri dishes. The next day, the cells were harvested after fixing with 4% paraformaldehyde for 2 min. Subsequently, a 2.5% electron microscope fixator (P1126; Solarbio, Beijing, China) was added to the cell pellet. Finally, the autophagosomes were observed and counted using a transmission electron microscope (TECNA I20; Philips, Eindhoven, Netherlands).

### Statistical Analysis

SPSS 22.0 software (SPSS, Inc., Chicago, IL, USA), GraphPad Prism 7.0, and R version 3.6.1 were used for the statistical analysis. Continuous variables were presented as the mean ± standard error. Student’s *t*-test or one-way ANOVA was used to evaluate differences in the experimental data. The relationship between ATF4 expression and clinicopathological indicators was analyzed using the chi-square test. The Kaplan–Meier method and Cox proportional hazards regression were used for survival analysis and nomogram construction, respectively. Correlation analysis was performed to evaluate the correlation between the expressions of ATF4 and ASNS. Statistical significance was set at *p* ≤ 0.05 (*p < 0.05; **p < 0.01; ***p < 0.001).

## Results

### ATF4 Expression Increases in GC Cells and Tissues and Primarily Localizes to the Nucleus

**Figure 1:**
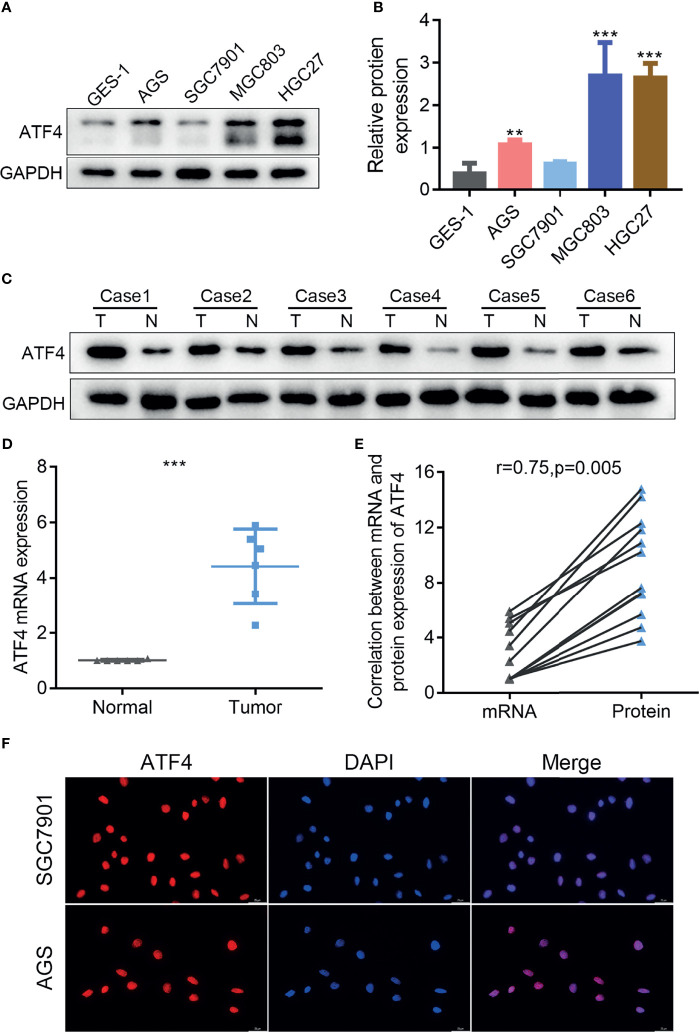
The expression of activating transcription factor 4 (ATF4) is elevated in gastric cancer (GC) cells and tissues and localizes to the nucleus. **(A)** Western blotting was used to measure the protein level of ATF4 in the GES-1 and GC cell lines. **(B)** Relative amount of ATF4 protein is presented as the mean ± SD of three experimental replicates (unpaired *t*-test: *p* < 0.05). **(C, D)** The ATF4 levels in tumor tissues (*T*) and corresponding nontumor tissues (*N*) from six GC patients were analyzed using immunoblotting and qRT-PCR (unpaired *t*-test: *p* = 0.0001). **(E)** Correlation analysis between the mRNA and protein expressions of ATF4 in six pairs of GC tissues (Pearson’s correlation analysis: *r* = 0.75, *p* = 0.005). **(F)** Immunofluorescence confirms the subcellular localization of ATF4 to the nucleus. **p < 0.01; ***p < 0.001.

The expression and intracellular localization of ATF4 in GC remain unclear. Therefore, we examined the expression of ATF4 in GC cells and tissues by WB and RT-PCR. We first examined expression levels of ATF4 in the normal gastric epithelial cell GES-1 and four GC cell lines (AGS, SGC7901, MGC803, and HGC27). The results showed that ATF4 expression in GC cells was significantly higher than that in GES-1 (**Figures 1A, B**). In addition, the amount of ATF4 was significantly upregulated in GC tissues compared with the corresponding normal tissues (**Figures 1C, D**). We quantified the bands in **Figure 1C** and conducted a correlation analysis between the expressions of mRNA and protein in GC tissues. The results showed that the expressions of ATF4 mRNA and protein were significantly consistent in the same batch of GC tissues (**Figure 1E**). Thereafter, the subcellular localization of ATF4 was assessed by IF in AGS and SGC7901 cells. The results showed that ATF4 was localized to the nucleus, and its expression in AGS cells was higher than that in SGC7901 cells, which was consistent with the WB results (**Figure 1F**). Taken together, these results suggested that ATF4 expression was elevated in GC cells and tissues and the ATF4 protein localized to the nucleus.

### High ATF4 Expression Is Strongly Associated With Tumor Aggressiveness and Overall Survival in GC

**Figure 2:**
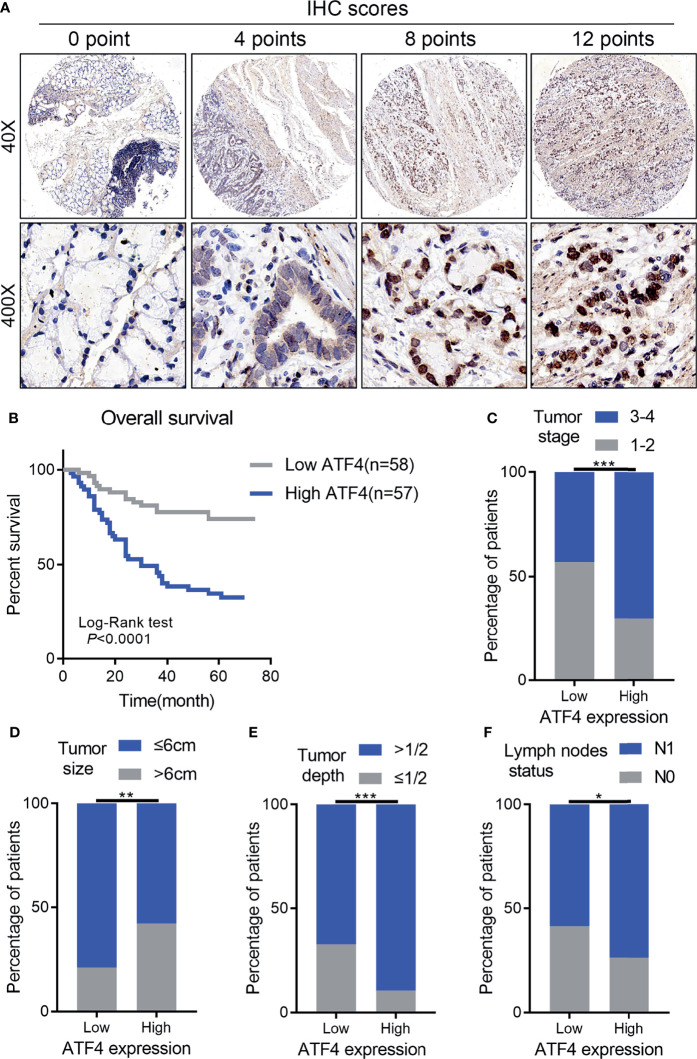
High activating transcription factor 4 (ATF4) expression is strongly associated with tumor aggressiveness and overall survival in gastric cancer (GC). **(A)** Representative images of ATF4 expression in tissue arrays of GC patients (*n* = 115). The final immunohistochemical (IHC) score was determined as the product of the staining intensity and the staining area. **(B)** Overall survival of GC patients with high and low ATF4 expressions (log-rank test: *p* < 0.0001). **(C–F)** Correlation between ATF4 expression and tumor stage, tumor size, tumor depth, and lymph node metastasis (chi-square test: *p* < 0.05). *p < 0.05; **p < 0.01; ***p < 0.001.

IHC for the TMA was performed to examine the correlation between ATF4 expression and GC aggressiveness. A total of 115 GC tissues with complete follow-up information were included in the final prognosis and clinical correlation analysis based on the evaluation of the expression levels of ATF4. The results indicated that GC tissues had increased levels of ATF4 as compared to normal tissues (**Figure 2A**). Kaplan–Meier analysis suggested that a low ATF4 expression presented a longer overall survival (OS) time (*p* < 0.001; **Figure 2B**). Furthermore, the chi-square test confirmed that the level of ATF4 was strongly associated with tumor stage, tumor size, tumor depth, and lymph node metastasis (*p* < 0.05; **Figures 2C**–**F**).

**Figure 3:**
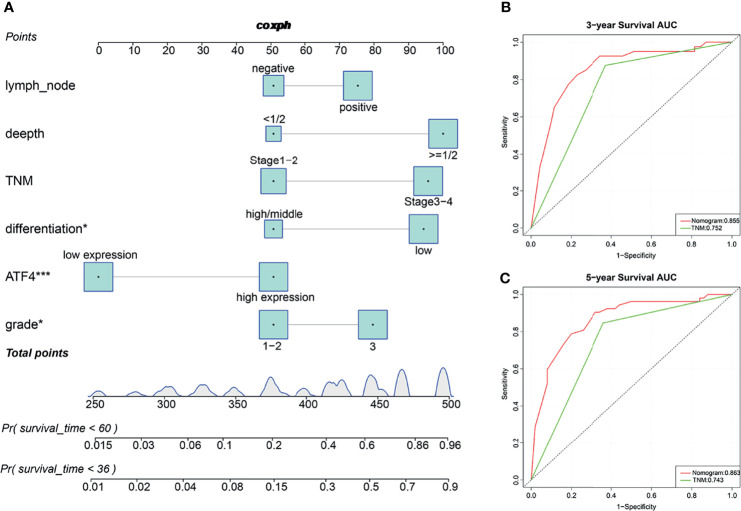
Predictive nomograms and receiver operating characteristic (ROC) curves of the 3- and 5-year overall survival (OS) of gastric cancer (GC) patients. **(A)** Predictive nomograms of the 3- and 5-year OS in tissue arrays of GC patients (*n* = 115). **(B)** ROC curves based on the nomograms of the 3-year OS in tissue arrays of GC patients (AUC = 0.855). **(C)** ROC curves based on the nomograms of the 5-year OS in tissue arrays of GC patients (AUC = 0.863). *AUC*, area under the ROC curve. *p < 0.05; ***p < 0.001.

Subsequently, the prognostic nomogram for OS was constructed based on Cox regression analysis. Univariate analysis indicated that the lymph node status, tumor depth, TNM stage, tumor differentiation, ATF4 expression, and tumor grade were significantly associated with the OS of GC patients (**Table 1**). All of these prognostic factors were further used for constructing the 3- and 5-year OS nomograms for GC (**Figure 3A**). The areas under the receiver operating characteristic curves (AUCs) for 3- and 5-year OS were 0.855 and 0.863, respectively (**Figures 3B, C**). Multivariate Cox regression analysis identified ATF4, tumor grade, and tumor differentiation as independent prognostic factors in GC (**Table 1**). Collectively, our data revealed that ATF4 expression was strongly associated with tumor aggressiveness and overall survival in GC patients.

### ATF4 Promotes GC Cell Proliferation, Migration, and Invasion *In Vitro*

**Figure 4:**
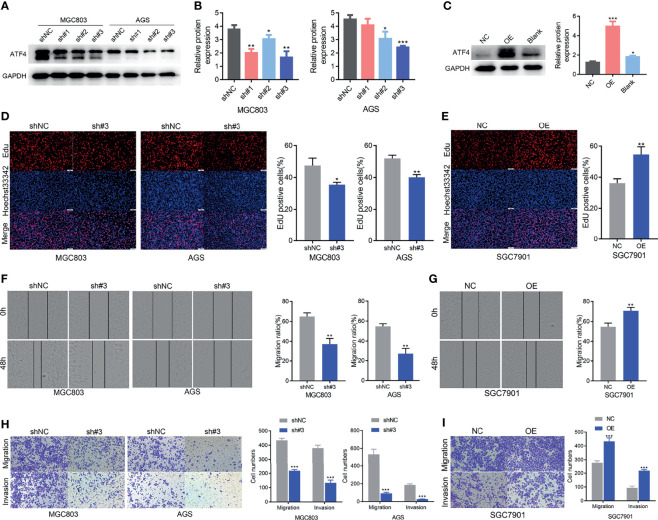
Activating transcription factor 4 (ATF4) promotes gastric cancer (GC) cell proliferation, migration, and invasion *in vitro*. **(A, B)** Western blotting was used to verify the efficiency of ATF4 knockdown. sh#3 showed a better knockdown efficiency. **(C)** Western blotting was used to verify the efficiency of ATF4 overexpression. **(D, E)** EdU assays for GC cells after ATF4 silencing and overexpression. The results suggested that cell growth was inhibited upon ATF4 knockdown in MGC803 (35.4% *vs*. 47.4%) and AGS cells (41.0% *vs*. 51.9%), while the overexpression of ATF4 exerted the opposite effects in SGC7901 cells (54.5% *vs*. 36.2%). **(F, G)** Cell migration in the MGC803, AGS, and SGC7901 cell lines after infection after scratch wound healing. Cell migration capacity was inhibited in MGC803 and AGS cells and was promoted in SGC7901 cells. **(H)** Transwell assays indicated that silencing ATF4 markedly decreases the invasion and vertical migration capacities of MGC803 (64.9% *vs*. 37.2%) and AGS cells (54.6% *vs*. 26.9%). **(I)** The invasion and vertical migration capacities in SGC7901 cells were improved after ATF4 overexpression (54.5% *vs*. 70.6%). Unpaired *t*-tests were used. *p < 0.05; **p < 0.01; ***p < 0.001.

To elucidate the function of ATF4 in GC, three lentiviral shRNAs were infected in AGS and MGC803 cell lines to knock down endogenous ATF4. An overexpression lentivirus of ATF4 was also used to infect SGC7901 cells. The knockdown and overexpression efficiencies are shown in **Figures 4A**–**C**. sh#3 was selected for further experiments due to its higher inhibition rates. We first evaluated the changes in the cell proliferation rates using the EdU assay, and the results suggested that GC cell growth was significantly inhibited upon ATF4 knockdown (**Figure 4D**). Wound-healing assays were also conducted in GC cells. The results showed that knocking down ATF4 could significantly inhibit the horizontal migration rate in MGC803 and AGS cells at 48 h (64.9% *vs*. 37.2% and 54.6% *vs*. 26.9%, respectively; **Figure 4F**). The Transwell assays indicated that silencing ATF4 resulted in a marked decrease in the invasion and vertical migration capacities of MGC803 and AGS cells (**Figure 4H**). In addition, overexpression of ATF4 in SGC7901 cells exerted the opposite effects (**Figures 4E, G, I**). Taken together, ATF4 may promote GC cell proliferation, migration, and invasion *in vitro*.

### ATF4 Silencing Promotes Cell Death and Inhibits S-Phase Progression *In Vitro*

**Figure 5:**
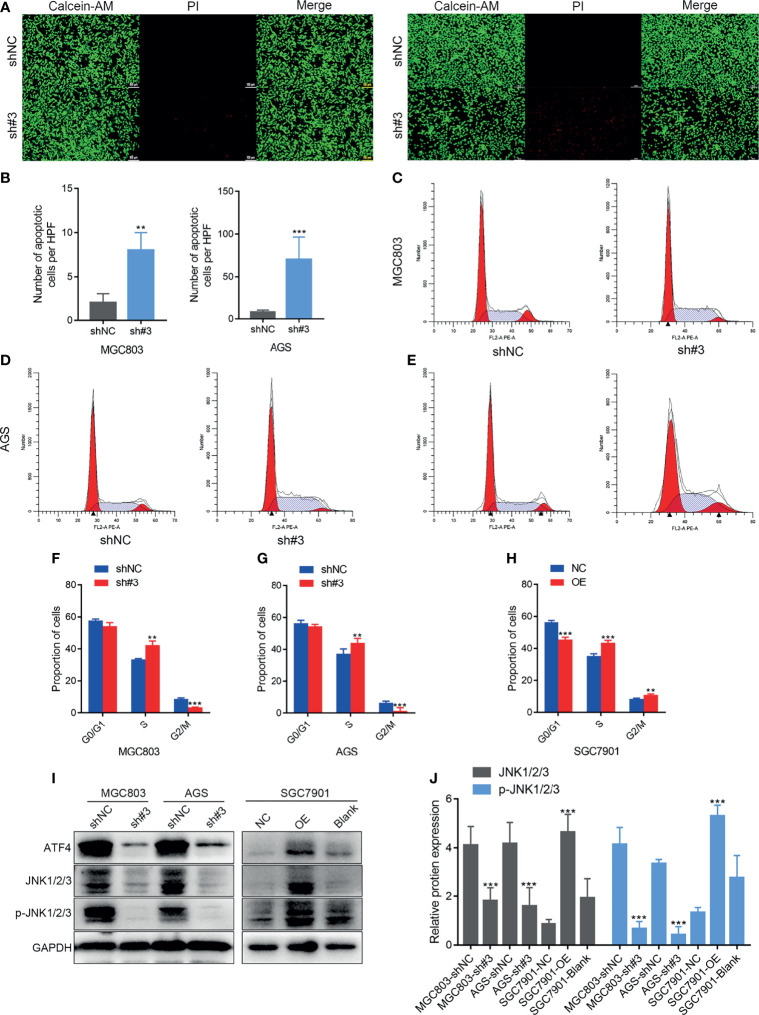
Activating transcription factor 4 (ATF4) silencing promotes cell death and inhibits S phase progress *in vitro*. **(A, B)** Calcein-AM/propidium iodide (PI) staining after shATF4 stable polyclonal MGC803 and AGS cells were established, respectively. **(C–H)** Cell cycle analysis for shATF4 and ATF4-WT lentivirus-infected MGC803, AGS, and SGC7901 cells after antibiotic selection for 2 weeks. **(C, D, F, G)** The proportion of S phase cells was increased and the proportion of G2M phase cells was decreased after ATF4 knockdown in MGC803 and AGS cells. **(E, H)** The proportion of G0/G1 phase cells was decreased, while the proportions of S and G2M phase cells were increased after ATF4 overexpression. **(I, J)** Relative protein expressions of JNK1/2/3 and p-JNK1/2/3 after ATF4 knockdown and overexpression. Unpaired *t*-tests were used. **p < 0.01; ***p < 0.001.

Calcein-AM/PI staining and flow cytometry were performed to examine whether ATF4 could regulate GC cell death and the cell cycle. Apoptotic cells could be stained red by PI. ImageJ software was used to count the cells that displayed red fluorescence. The results of Calcein-AM/PI staining demonstrated that ATF4 knockdown could markedly increase apoptosis in GC cells (**Figures 5A, B**). The Calcein-AM staining intensity in the ATF4 small hairpin RNA-3 (shATF4) group was also slightly weaker compared to that in the normal control of small hairpin RNA (shNC) group, which suggested that cell proliferation activity was suppressed upon knockdown. Flow cytometry analysis showed that the proportion of S phase cells was increased and the proportion of G2M phase cells was decreased after ATF4 knockdown (**Figures 5C, D, F, G**). On the other hand, in SGC7901 cells, the proportion of G0/G1 phase cells was decreased and that of S and G2M phase cells was increased after ATF4 overexpression (**Figures 5E, H**). These results suggested that ATF4 could promote cell cycle progression. To understand the mechanism underlying ATF4 regulation of the cell cycle, we examined the expression level of c-Jun N-terminal kinase (JNK1/2/3) after ATF4 knockdown and overexpression. JNK1/2/3 plays a crucial role in regulating the GC cell cycle and apoptosis (27). In addition, several studies have shown that JNK1/2/3 serves as an upstream of ATF4 (28, 29). Our results suggested that ATF4 could also affect the expression levels of JNK1/2/3 and p-JNK1/2/3 (**Figures 5I, J**). Taken together, these results indicated that ATF4 may promote S phase progression and inhibit cell death, possibly in a JNK-dependent manner in GC cells.

### ATF4 Promotes GC Progression, Possibly *via* Regulating Amino Acid Metabolism and Autophagy Pathways

**Figure 6:**
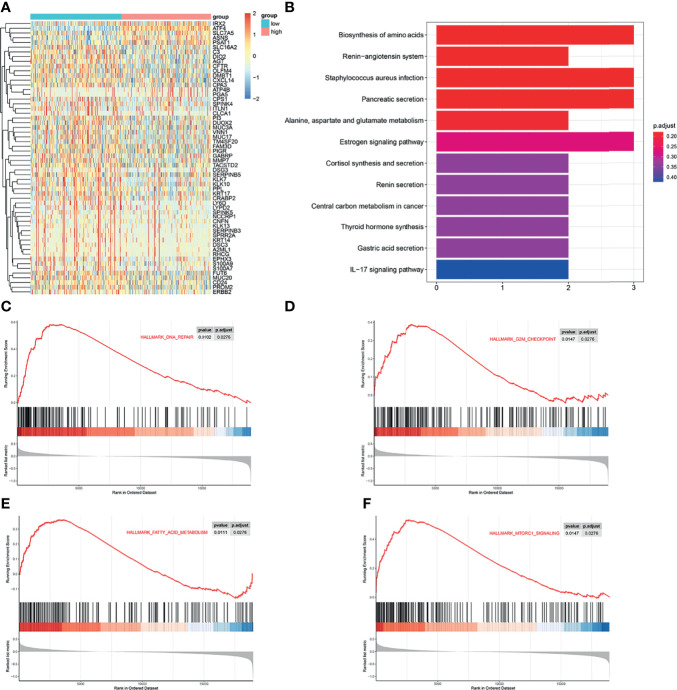
Activating transcription factor 4 (ATF4) promotes gastric cancer (GC) progression possibly *via* regulating the amino acid metabolism and autophagy pathways. **(A)** Heatmap of the differentially expressed genes in GC patients with different expression levels of ATF4. **(B)** Kyoto Encyclopedia of Genes and Genomes (KEGG) analysis was used to identify the significantly enriched biological processes associated with the differentially expressed genes in GC patients. **(C–F)** Gene set enrichment analysis (GSEA) showed that ATF4 may be related to DNA repair, G2M checkpoint, mTORC1 pathway, and amino acid metabolism processes.

To further investigate the function of ATF4 in GC, the transcript information of GC patients from TCGA database was assessed for varying expression levels of ATF4. We first identified 58 genes most closely associated with ATF4 expression, including the gene encoding asparagine synthetase (*ASNS*) (**Figure 6A**). KEGG pathway enrichment analysis demonstrated that these differentially expressed genes were mainly enriched in the amino acid synthesis pathway (**Figure 6B**). The results of GSEA indicated that ATF4 could regulate DNA repair, G2M checkpoint, and amino acid synthesis, consistent with the results of the cell cycle and KEGG analyses (**Figures 6C**–**E**). Gene ontology (GO) enrichment analysis indicated that ATF4 could be related to autophagy in GC (**Figure S1**). GSEA showed that ATF4 could also regulate the mTORC1 pathway (**Figure 6F**). Previous studies suggested that mTORC1 is involved in autophagy (30). Therefore, we reasonably hypothesized that ATF4 regulates autophagy, most likely through the mTORC1 pathway. Collectively, these results indicated that ATF4 could promote GC progression possibly by regulating the amino acid metabolism and autophagy pathways.

### ATF4 May Regulate Asn Metabolism by Promoting ASNS Expression in GC

**Figure 7:**
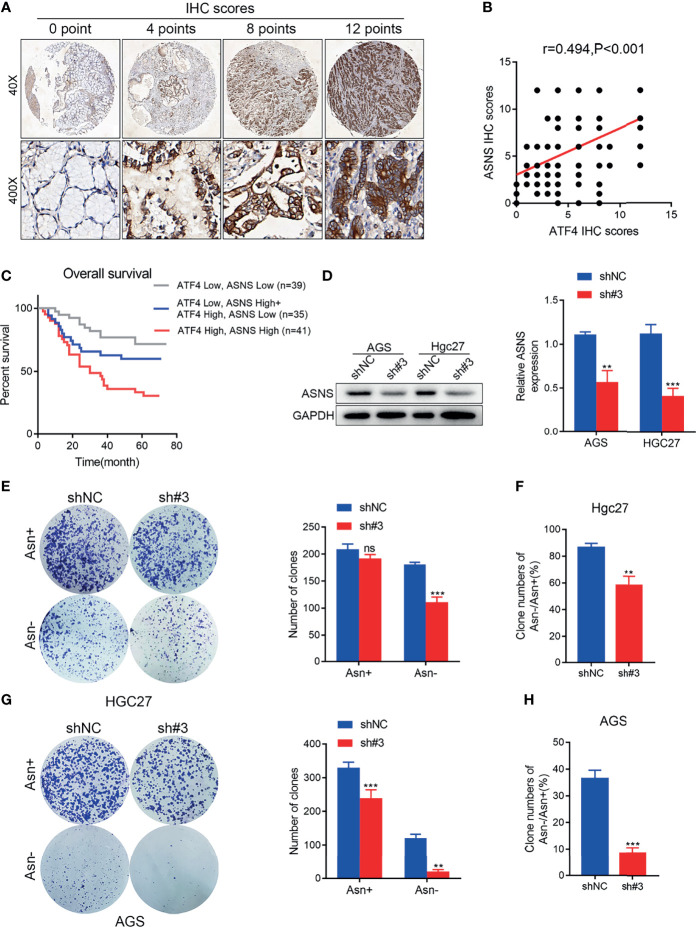
Activating transcription factor 4 (ATF4) may regulate asparagine (Asn) metabolism by promoting asparagine synthetase (ASNS) expression in gastric cancer (GC). **(A)** Representative images for ASNS expression in tissue arrays of GC patients (*n* = 115). The final immunohistochemical (IHC) score was determined as the product of the staining intensity and the staining area. **(B)** Pearson’s correlation analysis for the levels of ATF4 and ASNS proteins in patients with GC measured by IHC (*r* = 0.494, *p* < 0.001). **(C)** A combination of the expressions of ATF4 and ASNS can more accurately predict overall survival (OS) in GC patients. Patients with high expressions of both ATF4 and ASNS showed the worst survival status. **(D)** ATF4 silencing significantly inhibited the level of ASNS in AGS and HGC27 cells. **(E, G)** Clone formation assay in medium supplemented or deprived of Asn after ATF4 knockdown. The results confirmed that the inhibition of cell clone formation under Asn deprivation was more significant in the ATF4 interference group. **(F, H)** Statistical tests comparing the inhibition of colony forming ability in the control and ATF4 interference groups under Asn-deprived conditions. Unpaired *t*-tests were used. **p < 0.01; ***p < 0.001; *ns*, not significant.

To explore the clinical correlation between ATF4 and ASNS expressions, the level of ASNS was also evaluated in the same batch of tissue array (**Figure 7A**). Kaplan–Meier analysis indicated that a high level of ATF4 or ASNS in GC was substantially associated with poor OS (**Figure 7B**). Correlation analysis suggested that the IHC score for ATF4 staining was significantly positively correlated with that of the ASNS signal (*r* = 0.494, *p* < 0.001; **Figure 7C**). Subsequently, we examined the change in ASNS expression upon ATF4 knockdown. The results indicated that ATF4 silencing could significantly inhibit the level of ASNS in GC cells (**Figure 7D**). Based on the above results, we hypothesized that ATF4 was involved in Asn metabolism through regulating the expression of ASNS. To further verify this hypothesis, clonal formation experiments in medium with different concentrations of Asn (50 *vs*. 500 nM) were performed. The results confirmed that the inhibition of cell clone formation under Asn deprivation was more significant in the ATF4 interference group (**Figures 7E, G**). The statistical tests comparing the inhibition of colony forming ability in the control and ATF4 interference groups under Asn-deprived conditions are shown in **Figures 7F, H**. Taken together, ATF4 may regulate Asn metabolism through promoting ASNS expression in GC.

### Silencing ATF4 Inhibits Autophagy Through Regulating the mTORC1 Pathway in GC Cells

**Figure 8:**
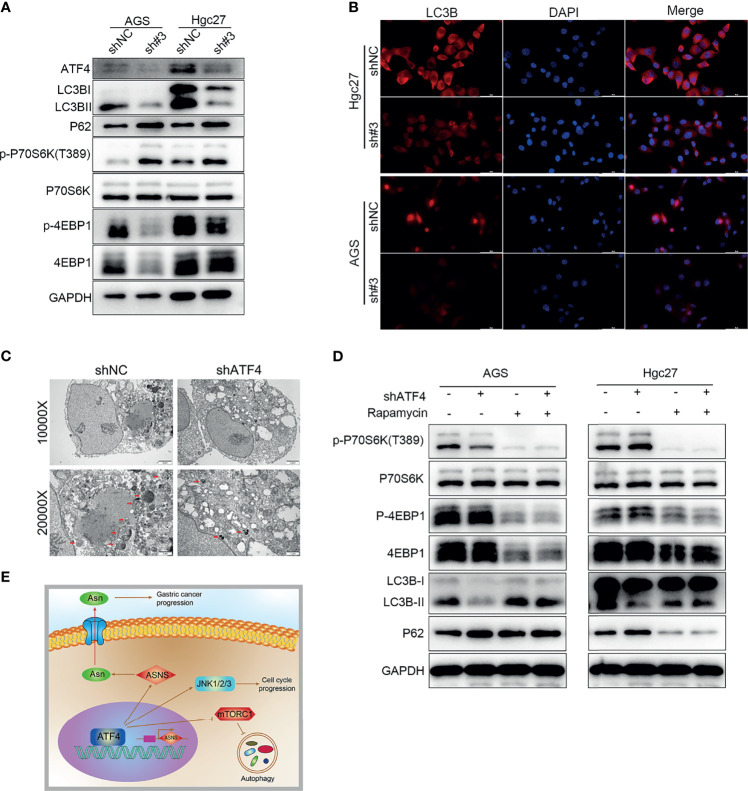
Silencing activating transcription factor 4 (ATF4) inhibits autophagy through regulating the mTORC1 pathway in gastric cancer (GC) cells. **(A)** Changes in LC3B, P62, p-P70S6K, and p-4EBP1 protein levels after ATF4 knockdown. **(B)** LC3B is detected by immunofluorescence (IF) after inhibition of ATF4 expression, and the level of LC3B was significantly decreased upon ATF4 knockdown. **(C)** TEM images showing that autophagic vacuoles in AGS cells decreased markedly upon ATF4 silencing. **(D)** Differences in the autophagy levels disappeared between the shATF4 and shNC groups after inhibition of the mTORC1 pathway. **(E)** Schematic depicting the role of ATF4 in GC. ATF4 may upregulate asparagine (Asn) metabolism by promoting asparagine synthetase (ASNS) expression, thereby facilitating GC progression; moreover, ATF4 can inhibit the mTORC1 pathway and subsequently suppress the level of autophagy in GC cells.

As mentioned above, ATF4 can regulate the mTORC1 pathway based on GSEA, and mTORC1 is involved in autophagy (31). Hence, we first detected the changes in the expression of the LC3B protein to verify whether ATF4 expression could regulate autophagy. The results of WB demonstrated that silencing ATF4 could significantly inhibit the levels of LC3B (**Figure 8A**). IF also confirmed that the proportion of perinuclear LC3B particles markedly decreased in the shATF4 group (**Figure 8B**). We next examined the phosphorylation levels of the downstream mTORC1 proteins P70S6K and 4EBP1. The amount of p-P70S6K significantly increased after ATF4 silencing, which suggested that the mTORC1 pathway was substantially activated (**Figure 8A**). We also found that the level of p-4EBP1 was decreased after ATF4 silencing, which was consistent with the results of a previous study (31). However, another study showed different results (32). As previously reported, mTORC1 was a negative regulator of 4EBP1 and should be downregulated after mTORC1 upregulation (33). The contradictory results may be due to 4EBP1 possibly being regulated by other proteins besides mTORC1, such as GSK3 and p38 (33). TEM images also showed that the autophagic vacuoles in GC cells were decreased markedly upon silencing ATF4 (**Figure 8C**). When the mTORC1 pathway was inhibited by rapamycin treatment after ATF4 knockdown, the significant differences in autophagy levels disappeared between the shATF4 and shNC groups (**Figure 8D**). The results also showed that the changes of P62 in AGS and HGC27 cells were incongruent (**Figure 8D**). This may be caused by the different sensitivity of AGS and HGC27 cells from that of rapamycin. Taken together, ATF4 could regulate autophagy through the mTORC1-mediated pathway in GC.

## Discussion

ATF4 plays vital roles in several human cancer types, such as breast cancer, colon cancer, and prostate cancer (34–36). However, all these studies emphasized ATF4 as a downstream molecule rather than the starting point of the cascade of signaling events. Moreover, no study has comprehensively evaluated the effects of ATF4 in GC. Therefore, we examined the mechanism underlying ATF4 regulation of GC progression systematically by detecting its expression, cellular localization, and influence on prognosis and cell function. The results of this study are summarized as a schematic shown in **Figure 8E**.

It has been previously reported that ATF4 was overexpressed and primarily localized in the nucleus in lung cancer cells (37). In the present study, we performed RT-PCR, WB, and IF experiments. The results showed that ATF4 expression was upregulated in GC cells and that the protein was localized to the nucleus (**Figure 1**). Subsequently, we demonstrated that ATF4 was associated with the prognosis of GC by evaluating the expression levels and analyzing the self-generated TMAs (**Figure 2** and **Table 1**). Moreover, we constructed an accurate nomogram model for predicting the prognosis of GC based on ATF4 expression (**Figure 3**). There are only a few studies on the role of ATF4 in tumor prognosis, and the results remain controversial (38, 39). In triple-negative breast cancer, a high ATF4 expression was shown to be correlated with low OS after diagnosis (37 months for high ATF4 expression and 46 months for low ATF4 expression); however, the difference between the two groups was not statistically significant (*p* = 0.125) (38). Rozita et al. raised a different point of view (39). They identified ATF4 as a negative regulator in medullary thyroid cancer, and a low ATF4 expression was associated with worse OS in medullary thyroid cancer patients (39). These findings suggest that ATF4 may play a dual role in different cancer types. However, in GC, ATF4 could significantly promote tumor progression and was negatively correlated with the prognosis.

Next, we studied the function of ATF4 in GC cells through wound-healing, Transwell, and EdU assays and Calcein-AM/PI staining. Briefly, overexpressing and silencing ATF4 significantly influenced cell proliferation, invasion, migration, and cell death (**Figures 4** and **5A, B**). Previous studies also demonstrated that the inhibition of ATF4 expression reduces cell migration, invasion, and proliferation in breast cancer (38). Wu et al. reported that siRNA against ATF4 leads to G1 phase arrest in chondrogenic cells (40). However, our cell cycle assays suggested that ATF4 knockdown resulted in an arrested cell cycle in the S phase, while the overexpression of ATF4 exerted the opposite effect (**Figures 5C**–**J**). To further support these findings, we conducted the GSEA and found that ATF4 was involved in the regulation of DNA repair and G2/M checkpoint (**Figures 6C, D**). Therefore, a decrease in ATF4 expression may affect DNA synthesis in the S phase and the G2M phase transition. Subsequently, we analyzed the RNA sequencing data of GC patients in TCGA database and found that ATF4 was closely related to the amino acid metabolism and autophagy pathways (**Figures 6A, B** and **Supplementary Figure S1**). Thus, we further performed the relevant experiments to verify these preliminary data.

According to the analysis of TCGA data, ATF4 could promote Asn metabolism by enhancing the transcription of ASNS. A previous study also demonstrated that ASNS was recognized by ATF4 and contributes to protein biosynthesis in lung cancer (41). Therefore, we examined the expression of ASNS in the same batch of GC tissue microarrays (**Figure 7A**). Survival analysis indicated that the combination of the expression levels of AFT4 and ASNS exhibited a more significant predictive accuracy for GC prognosis (**Figure 7C**). ASNS expression was suppressed upon ATF4 knockdown, which proved that these two proteins could interact (**Figure 7D**). Furthermore, clonal formation experiments after Asn deprivation also showed that ATF4 prominently affected Asn metabolism (**Figures 7E**–**H**). Taken together, our results demonstrated one of the functional mechanisms of ATF4 in GC.

Finally, we explored the relationship between ATF4 and autophagy. GO enrichment analysis and GSEA showed that ATF4 was closely related to autophagy and the mTORC1 pathway, respectively (**Figure 8**). Combined with the results of previously published literature, mTORC1 activation suggested that autophagy was inhibited in cells (42, 43). Hence, we reasonably hypothesized that ATF4 could also regulate autophagy through the mTORC1 pathway in GC; further experimental results confirmed this hypothesis. A previous study demonstrated that ATF4 overexpression conferred the multidrug resistance phenotype to GC cells (44). Therefore, ATF4 may play multiple roles in GC and is worthy of further investigation.

In conclusion, our study is the first to comprehensively assess the expression, clinical significance, and function of ATF4 in GC. Our results indicated that ATF4 was overexpressed in GC and was associated with poor OS in GC patients. ATF4 significantly promoted the migration, invasion, proliferation, and cell cycle progression *in vitro*. Mechanistically, ATF4 promoted GC progression, possibly through regulating the amino acid metabolism and autophagy pathways. Future research should focus on the efficacy of targeted ATF4 therapy in GC treatment.

## Data Availability Statement

The original contributions presented in the study are included in the article/**Supplementary Material**. Further inquiries can be directed to the corresponding author.

## Ethics Statement

The studies involving human participants were reviewed and approved by Anhui Medical University Ethics Committee. The ethical code is 20180323. The patients/participants provided written informed consent to participate in this study.

## Author Contributions

MW and YLi conceived the study design. MW wrote the manuscript. MW and YLu performed the experiments. MW, HW, and YLu were involved in the cell culture and *in vitro* experiments. YW and XX were responsible for clinical sample collection. All authors contributed to the article and approved the submitted version.

## Funding

This research was funded by the National Natural Science Foundation (grant no. 81874063).

## Conflict of Interest

The authors declare that the research was conducted in the absence of any commercial or financial relationships that could be construed as a potential conflict of interest.

## Publisher’s Note

All claims expressed in this article are solely those of the authors and do not necessarily represent those of their affiliated organizations, or those of the publisher, the editors and the reviewers. Any product that may be evaluated in this article, or claim that may be made by its manufacturer, is not guaranteed or endorsed by the publisher.
