# Supplementary material for: High ATF4 Expression Is Associated With Poor Prognosis, Amino Acid Metabolism, and Autophagy in Gastric Cancer
Source: Front Oncol. 2021 Dec 17;11:740120. doi: 10.3389/fonc.2021.740120 (PMC8718699; doi:10.3389/fonc.2021.740120)
Supplement: Supplementary file 1 [file DataSheet_1.docx]

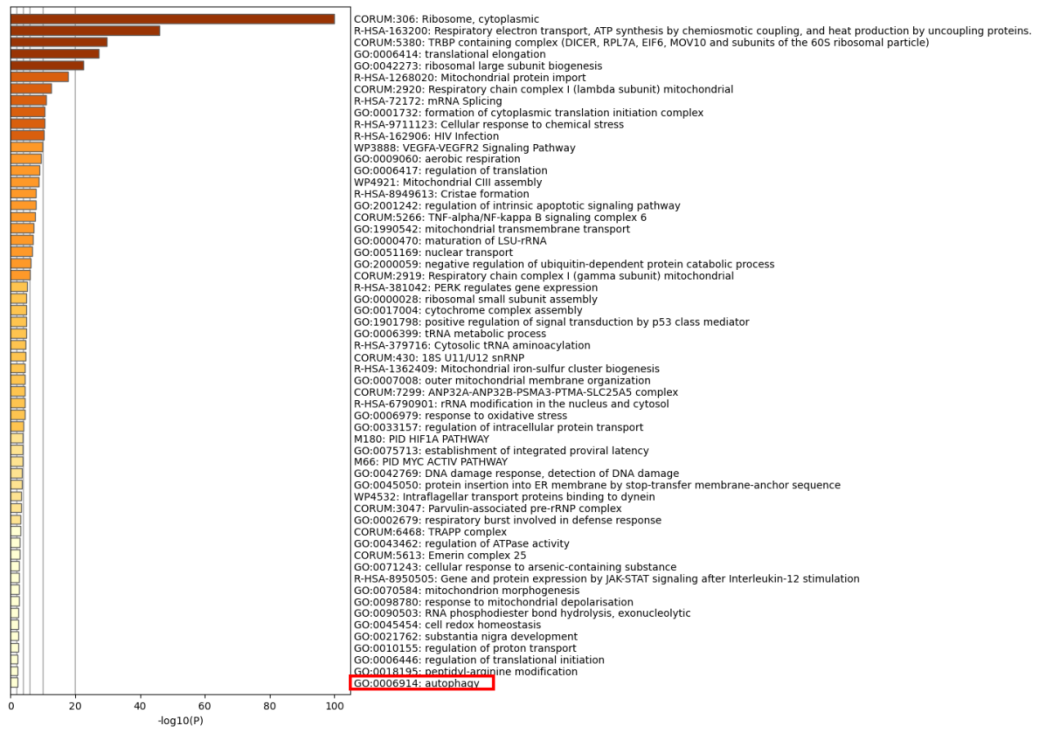


**Figure S1:** Gene ontology enrichment analysis indicated that ATF4 may be related to autophagy in GC.
